# Clinical efficacy and safety of first‐line nilotinib or imatinib therapy in patients with chronic myeloid leukemia—Nationwide real life data

**DOI:** 10.1002/cam4.70158

**Published:** 2024-09-13

**Authors:** Petra Belohlavkova, Daniela Zackova, Hana Klamova, Edgar Faber, Michal Karas, Lukas Stejskal, Eduard Cmunt, Olga Cerna, Ivana Jeziskova, Katerina Machova Polakova, Pavel Zak, Tereza Jurkova, Marika Chrapava, Jiri Mayer

**Affiliations:** ^1^ 4th Department of Internal Medicine and Haematology University Hospital Hradec Kralove and Charles University Prague Czech Republic; ^2^ Department of Internal Medicine–Haematology and Oncology University Hospital Brno and Masaryk University Brno Czech Republic; ^3^ Institute of Haematology and Blood Transfusion Prague Czech Republic; ^4^ Department of Haemato‐oncology University Hospital Olomouc and Palacky University Olomouc Czech Republic; ^5^ Department of Haemato‐oncology University Hospital Plzen and Charles University Plzen Czech Republic; ^6^ Department of Haemato‐oncology University Hospital Ostrava and Ostrava University Ostrava Czech Republic; ^7^ 1st Department of Internal Medicine—Haematology General University Hospital and Charles University Prague Czech Republic; ^8^ Department of Internal Medicine—Haematology University Hospital Kralovske Vinohrady and Charles University Prague Czech Republic; ^9^ Institute of Biostatistics and Analyses Masaryk University Brno Czech Republic; ^10^ Central European Institute of Technology (CEITEC) Masaryk University Brno Czech Republic

**Keywords:** chronic myeloid leukemia, first‐line treatment, imatinib, nilotinib

## Abstract

**Background:**

To evaluate the outcomes of first‐line imatinib versus nilotinib treatment for chronic myeloid leukemia in the chronic phase (CML‐CP) in real‐world clinical practice.

**Methods:**

A propensity score analysis was performed to eliminate imbalances between the treatment groups. In the analysis, 163 patients in the nilotinib group and 163 patients in the matched imatinib group were retrospectively evaluated.

**Results:**

Nilotinib‐treated patients achieved complete cytogenetic response (CCyR) and major molecular response more rapidly than imatinib‐treated patients. However, there was no significant difference in 5‐year overall survival (OS) or progression‐free survival (PFS) between the two groups (OS: 94.3% vs. 90.5%, *p* = 0.602; PFS: 92.9% vs. 88.0%, *p* = 0.614). Nilotinib‐treated patients had a higher failure‐free survival (FFS) and event‐free survival (EFS) than imatinib‐treated patients (FFS: 71.7% vs. 54.3%, *p* = 0.040; EFS: 71.7% vs. 53.5%, *p* = 0.025).

**Conclusions:**

This retrospective analysis from clinical practice did not confirm any benefit of frontline nilotinib treatment for OS and PFS; however, it did demonstrate higher FFS and EFS in the nilotinib cohort.

## 1 INTRODUCTION

The discovery of tyrosine kinase inhibitors (TKIs) has revolutionized the prognosis of patients with chronic myeloid leukemia in the chronic phase (CML‐CP). Before the advent of targeted therapy, patients with CML‐CP were commonly treated with interferon‐α (IFN‐α) plus cytarabine, which resulted in 3‐ to 5‐year overall survival (OS) rates ranging from 86% to 68%.^1^, ^2^ Imatinib, approved for clinical practice in 2001 for the treatment of all stages of CML, was the first TKI to be developed. An update of the International Randomized Study of Interferon and STI571 (IRIS) showed that the estimated 10‐year OS of imatinib‐treated patients was 83.3%.^3^, ^4^ In the ensuing years, the more efficacious second‐generation tyrosine kinase inhibitors (2GTKIs) dasatinib, nilotinib, and bosutinib were discovered. Clinical trials investigating the use of first‐line 2GTKIs have shown that dasatinib, nilotinib, and bosutinib induce a more rapid cytogenetic and molecular response in patients (DASISION, ENESTnd, and BFORE studies). Furthermore, the ENESTnd study also shows a lower incidence of progression to accelerated (AP) or blast phase (BP) chronic myeloid leukemia (CML).^5^, ^6^, ^7^, ^8^, ^9^, ^10^, ^11^, ^12^, ^13^, ^14^ However, the impact of utilizing 2GTKIs as first‐line therapy on patient OS and progression‐free survival (PFS) has not yet been definitively established in studies and remains a topic of debate. Consequently, treatment guidelines do not specify which of these medications should be used as first‐line therapy, and defining the optimal first‐line TKI treatment is still a significant challenge.^15^, ^16^, ^17^, ^18^

Patient age, comorbidities, biological features of CML‐CP, and the efficacy and safety of TKIs must always be considered when selecting first‐line therapy. Given the median age of CML diagnosis (59 years in the United States; 56 years in Europe) and the presence of comorbidities, imatinib remains the most common first‐line treatment choice.^19^, ^20^ In contrast, 2GTKIs are a more suitable first‐line treatment option for young patients with intermediate‐ or high‐risk disease due to their potency.^21^, ^22^, ^23^ Additionally, young women who plan to become pregnant represent another target group for first‐line treatment with 2GTKIs.^24^, ^25^, ^26^, ^27^, ^28^

The adverse event profiles of second‐generation TKIs differ due to variations in their off‐target effects. Nilotinib has been associated with cytopenias, hepatotoxicity (hyperbilirubinemia, increased serum alanine, or aspartate aminotransferase), abdominal pain, vomiting, nausea, diarrhea, or pancreatitis. Other very common side effects include dizziness, fatigue, musculoskeletal pains or muscle spasms, headache, and dermatologic problems. A large proportion of patients also experience metabolic and endocrinological changes such as hyperglycemia, hypophosphatemia, and increased HDL and LDL cholesterol. Nilotinib should be used with caution in patients who have or are at significant risk of developing QTc prolongation. Nilotinib can also lead to adverse effects different from those of imatinib, such as cardiovascular and cerebrovascular events. It can cause accelerated atherosclerosis and arterial thrombotic events (myocardial ischemia, stroke, and peripheral artery obstructive disease), with the risk increasing with the duration of nilotinib administration. It remains unclear whether these events during nilotinib treatment are primarily due to TKI‐induced metabolic comorbidities or the drug itself.^29^, ^30^, ^31^, ^32^, ^33^, ^34^, ^35^, ^36^

Despite the widespread use of nilotinib in clinical trials, there remains a paucity of data regarding its efficacy and safety as a first‐line treatment in real‐world clinical practice. Population‐based studies, which include all cases occurring within a defined region, provide valuable insights into the effectiveness of treatments in routine clinical settings without patient selection.^37^

In the Czech Republic, treatment of CML patients is centralized in eight specialized hematology centers. Patient data are collected and maintained in the INFINITY (*Tyrosine Kinase Inhibitors in FIrst and FollowIng CML Treatment*) database. This unique resource provides an opportunity to compare the outcomes of first‐line imatinib and nilotinib treatment in CML‐CP patients.

## 2 MATERIALS AND METHODS

### 2.1 Patients

In a retrospective analysis, we evaluated data from 984 patients treated for CML‐CP in the nationwide INFINITY database. Of these patients, 821 (83.4%) had received first‐line imatinib therapy, while 163 (16.6%) had initiated treatment with nilotinib. To mitigate the influence of age, sex, performance status, disease stage, and comorbidities, each patient in the nilotinib cohort was matched to a patient in the imatinib cohort using propensity score matching (Table 1). In the analysis, 163 patients in the nilotinib group, 163 patients in the matched imatinib group, and 658 patients in the unmatched imatinib group were retrospectively evaluated. Prior to matching, the groups were compared using Fisher's exact test for categorical variables or the non‐parametric Mann–Whitney test for continuous variables. All patients provided informed consent for data collection in the INFINITY registry by signing a consent form.

**TABLE 1:** Patient demographics and baseline characteristics.

Characteristics	Imatinib all (*n* = 821)	Nilotinib (*n* = 163)	*p*‐value	Imatinib matched (*n* = 163)	Imatinib unmatched (*n* = 658)	*p*‐value
Age at diagnosis years						
Median (min–max)	62.0 (18.0–91.0)	46.0 (18.0–80.0)	<0.001	46.0 (18.0–80.0)	64.0 (24.0–91.0)	<0.001
Mean (SD)	58.5 (15.1)	46.0 (14.2)		45.9 (15.5)	61.6 (13.3)	
Sex, *n* (%)						
Female *n* (%)	398 (48.5)	69 (42.3)	0.170	72 (44.2)	326 (49.5)	0.222
Male *n* (%)	423 (51.5)	94 (57.7)		91 (55.8)	332 (50.5)	
Sokal risk group *n* (%)						
Low risk	272 (33.1)	56 (34.4)	0.131	60 (36.8)	212 (32.2)	0.008
Intermediate risk	366 (44.6)	59 (36.2)		55 (33.7)	311 (47.3)	
High risk	163 (19.9)	44 (27.0)		44 (27.0)	119 (18.1)	
Unknown	20 (2.4)	4 (2.5)		4 (2.5)	16 (2.4)	
ELTS risk group *n* (%)						
Low risk	412 (50.2)	90 (55.2)	0.136	94 (57.7)	318 (48.3)	0.018
Intermediate risk	263 (32.0)	38 (23.3)		36 (22.1)	227 (34.5)	
High risk	125 (15.2)	31 (19.0)		29 (17.8)	96 (14.6)	
Unknown	21 (2.6)	4 (2.5)		4 (2.5)	17 (2.6)	
ECOG performance status *n* (%)						
0	444 (54.1)	114 (69.9)	<0.001	115 (70.6)	329 (50.0)	<0.001
1	274 (33.4)	42 (25.8)		42 (25.8)	232 (35.3)	
2	54 (6.6)	0 (0.0)		0 (0.0)	54 (8.2)	
3	12 (1.5)	1 (0.6)		1 (0.6)	11 (1.7)	
4	5 (0.6)	2 (1.2)		1 (0.6)	4 (0.6)	
Unknown	32 (3.9)	4 (2.5)		4 (2.5)	28 (4.3)	
Cerebrovascular disease *n* (%)						
Yes	37 (4.5)	0 (0.0)	0.002	0 (0.0)	37 (5.6)	<0.001
No	784 (95.5)	163 (100.0)		163 (100.0)	621 (94.4)	
Diabetes mellitus *n* (%)						
Yes	174 (21.2)	5 (3.1)	<0.001	2 (1.2)	172 (26.1)	<0.001
No	647 (78.8)	158 (96.9)		161 (98.8)	486 (73.9)	
Hyperlipidemia *n* (%)						
Yes	157 (19.1)	16 (9.8)	0.003	15 (9.2)	142 (21.6)	<0.001
No	664 (80.9)	147 (90.2)		148 (90.8)	516 (78.4)	
Ischemic cardiac disease *n* (%)						
Yes	92 (11.2)	2 (1.2)	<0.001	4 (2.5)	88 (13.4)	<0.001
No	729 (88.8)	161 (98.8)		159 (97.5)	570 (86.6)	

Abbreviations: ECOG, Eastern Cooperative Oncology Group; ELTS, the EUTOS long‐term survival score.

### 2.2 Statistical analysis

Valid ELN recommendations were used to evaluate the treatment response and treatment changes. All treatment responses for statistical analysis were defined according to Guilhot et al.^38^ If patients were discontinued deliberately, that is, for the study, to achieve a TFR, or because of pregnancy, these events were taken as a competitive risk at the date of discontinuation to avoid biasing the results. Survival probabilities were estimated using the Kaplan–Meier method. The Breslow test was used to compare patient survival between groups. Performance status was evaluated according to the Eastern Cooperative Oncology Group (ECOG) scale, and patients were classified according to their functional impairment.^39^ TKI toxicity was assessed using the Common Terminology Criteria for Adverse Events v4.0.^40^

## 3 RESULTS

### 3.1 Patients

A total of 984 CML‐CP patients were retrospectively evaluated from the INFINITY registry. Of these patients, 821 (83.4%) had received first‐line imatinib therapy, while 163 (16.6%) had initiated treatment with nilotinib. The median age of all patients treated with imatinib was 62.0 years (range 18.0–91.0 years), compared to the median age of 46.0 years (range 18.0–80.0 years) in the nilotinib group (*p* < 0.001). There were numerical differences in the proportion of patients with high‐risk prognostic scores according to the Sokal and the EUTOS long‐term survival score (ELTS) in the nilotinib group (Table 1). Nevertheless, patients treated with nilotinib had better overall performance status, with 69.9% of patients having an ECOG performance status of 0, compared to 54.1% of matched imatinib patients (*p* < 0.001). Furthermore, patients treated with nilotinib had significantly fewer comorbidities, including cerebrovascular disease, diabetes mellitus, hyperlipidemia, and ischemic cardiac disease (*p* = 0.002; *p* < 0.001; *p* = 0.002; and *p* < 0.001, respectively) (Table 1).

### 3.2 Response to treatments in the matched cohorts

Treatment with the standard dose of 400 mg/day was initiated in 97.5% (*n* = 163) of patients in the imatinib group, and the initial dose of 600 mg/day nilotinib was administered in 95.1% (*n* = 163) of patients (Table 2). Imatinib dose modification was necessary in 45 of 163 patients (27.6%): dose reduction in 34 of 163 (20.8%) and dose increase in 11 of 163 (6.7%). Imatinib treatment was discontinued in 79 of 163 patients (48.5%), with a median duration of treatment of 10 months (range, 0.7–99.2 months). The most common reason for imatinib discontinuation was treatment failure, observed in 39 of 163 patients (23.9%). Among these, failure occurred in 20 patients (51.3%) within the first 12 months of treatment. Conversely, imatinib intolerance led to treatment change in only 17 patients (10.4%) (Table 2).

**TABLE 2:** Patients' background (imatinib matched patients and nilotinib patients).

Characteristics *n* (%)	Imatinib (*n* = 163; 50.0%)	Nilotinib (*n* = 163; 50.0%)
Follow up months		
Median (min–max)	58.5 (0.8–131.0)	55.3 (1.6–121.7)
Starting daily dose, *n* (%)		
400 mg/600 mg	159 (97.5%)	155 (95.1%)
< 400 mg/600 mg	4 (2.5%)	2 (1.2%)
> 400 mg/600 mg	0 (0.0%)	6 (3.7%)
Treatment discontinuation *n* (%)		
Yes	79 (48.5%)	75 (46.0%)
No	84 (50.3%)	88 (54.0%)
Reason for treatment discontinuation *n* (%)		
Resistance	39 (23.9%)	23 (14.1%)
Intolerance^a^	17 (10.4%)	32 (19.6%)
Other^b^	13 (8.0%)	7 (4.3%)
Death	4 (2.5%)	2 (1.2%)
Discontinuation of trial	8 (4.9%)	15 (9.2%)
Treatment change period due to resistance *n* (%)		
0–3 months	5 (12.8%)	4 (17.4%)
3–6 months	4 (10.2%)	3 (13.0%)
6–12 months	11 (28.2%)	6 (26.1%)
12–24 months	12 (30.8%)	5 (21.8%)
24–36 months	3 (7.7%)	2 (8.7%)
>36 months	4 (10.3%)	3 (13.0%)
Treatment change period due to intolerance *n* (%)		
0–3 months	8 (47.0%)	3 (9.4%)
3–6 months	2 (11.7%)	3 (9.4%)
6–12 months	5 (29.4%)	4 (12.5%)
12–24 months	1 (7.0%)	5 (15.6%)
24–36 months	1 (7%)	2 (6.2%)
>36 months	0 (0%)	15 (46.9%)
Patients' status *n* (%)		
Alive	144 (88.3%)	148 (90.8%)
Dead	16 (9.8%)	12 (7.4%)
Lost to follow up	3 (1.8%)	3 (1.8%)

^a^ Intolerance also occurred in some patients who discontinued treatment due to resistance. There were 2 (2.5%) patients treated with imatinib and 4 (5.3%) patients treated with nilotinib.

^b^ Other reasons for imatinib discontinuation were clinical trial–4, lost of follow‐up–3, oncological therapy for tumor–3, on the patient's request–2, gravidity–1. Other reasons for nilotinib discontinuation were gravidity–4, lost of follow‐up–1 and 2 patients had no other reason specified.

In the nilotinib group, dose modification was performed in 43 of 163 patients (26.3%): dose reduction in 39 of 163 (23.9%) and dose increase in 4 of 163 patients (2.5%). Nilotinib treatment had to be discontinued in 75 of 163 patients (46.0%). For the nilotinib cohort, the median treatment duration was 21.5 months (range: 0.4–103.7 months). The primary reason for discontinuation was intolerance or toxicity, affecting 32 of 163 patients (19.6%). In contrast to the imatinib cohort, toxicity in the nilotinib group occurred in 15 patients (46.9%) after 36 months of treatment, predominantly due to metabolic and vascular causes. Treatment failure with nilotinib led to discontinuation in 23 of 163 patients (14.1%), with 13 of these patients (56.5%) experiencing treatment failure within the first 12 months (Table 2).

A total of 28 patients died in these matched groups: 16 of 163 patients (9.8%) with imatinib and 12 of 163 patients (7.4%) with nilotinib. The most common causes of death in the imatinib group were non‐CML causes (8 of 16 patients; 50.0%). All these patients were in the chronic phase of CML. In three cases, death occurred due to progression to advanced stages of CML. Three patients died from CML treatment‐related causes, specifically hematopoietic stem cell transplantation (HSCT) complications: multi‐organ failure after HSCT (1 patient) and GvHD (graft‐versus‐host disease; 2 patients).

In contrast, in the nilotinib group, the most common cause of death was CML progression (7 of 12 patients; 58.3%). The blast phase of CML was present at the time of death in these six patients. Five patients (41.7%) in the nilotinib cohort died from non‐CML causes, including one case of cardiovascular disease. There were no CML treatment‐related deaths in nilotinib‐treated patients.

Our analysis shows that successful TFR was achieved in 16/163 patients (9.8%) in the imatinib cohort and 24/163 patients (14.7%) in the nilotinib cohort. At this time, we do not have further detailed evaluations of these patients.

### 3.3 Long‐term outcomes in the matched cohorts

**FIGURE 1:**
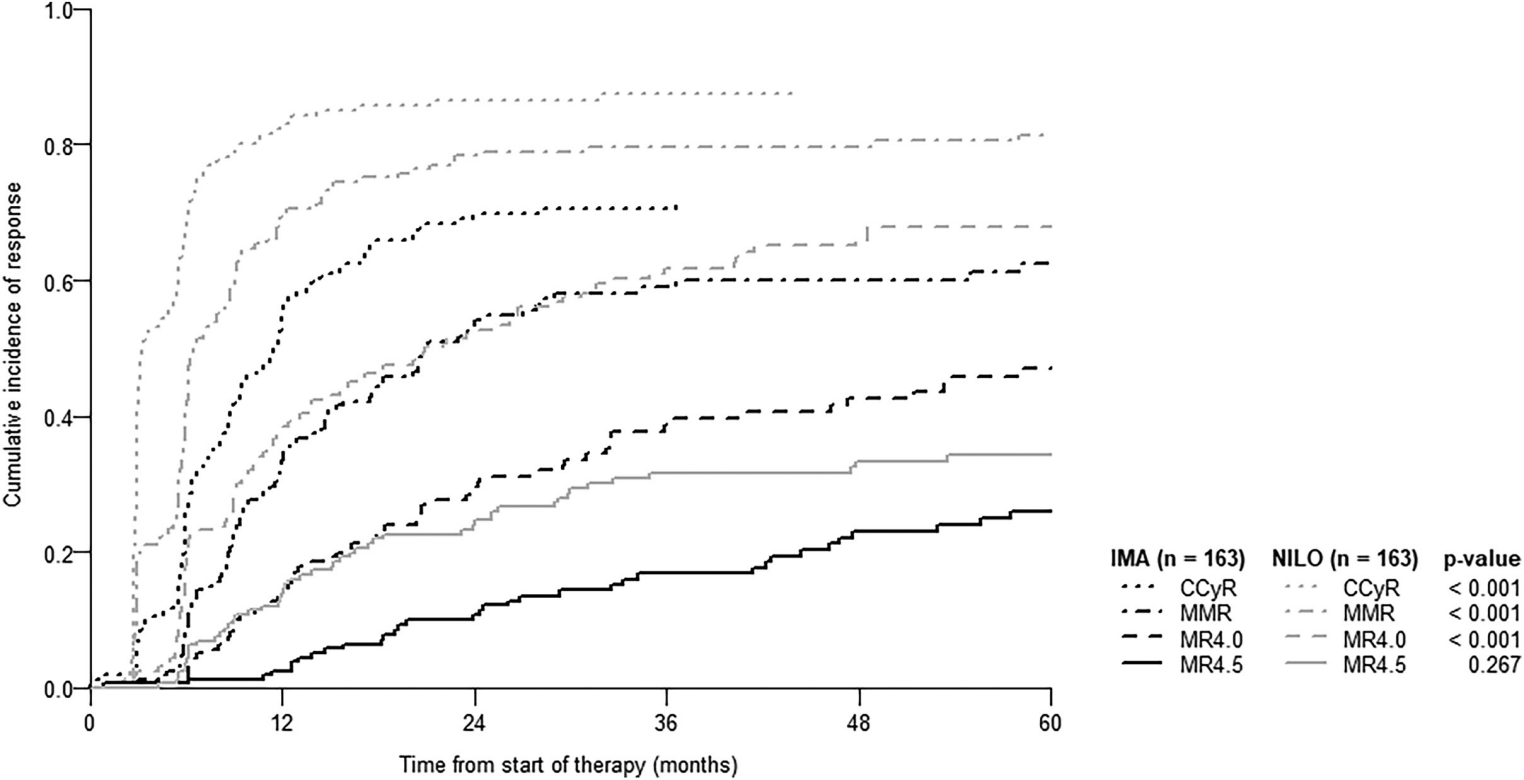
Cumulative incidence of response in imatinib or nilotinib cohorts after propensity matching.

The cumulative achievement of complete cytogenetic response (CCyR) and major molecular responses (MR^3^) or deeper favored frontline nilotinib treatment at all time points was found (Figure 1). At 6 and 12 months, CCyR rates were 32.9% and 62.5% in imatinib patients versus 74.3% and 81.2% in nilotinib patients, respectively (*p* < 0.001). The proportion of patients achieving MMR or deeper molecular responses in the imatinib matched versus the nilotinib cohorts was 15.7% versus 54.0%, respectively, at 6 months, and 42.5% versus 69.2%, respectively, at 12 months (*p* < 0.001).

**FIGURE 2:**
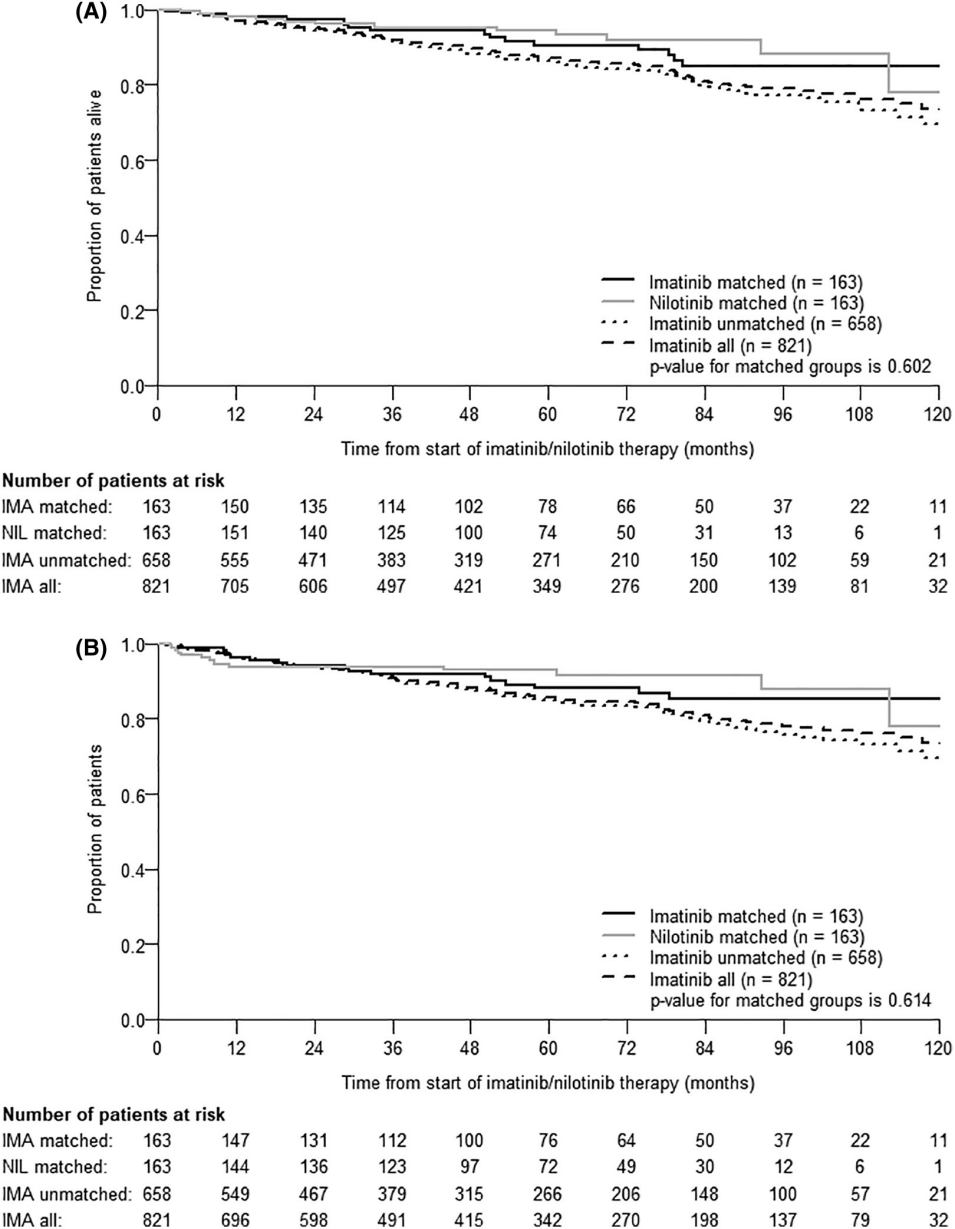
(A) Overall survival. (B) Progression‐free survival (survival analysis of matched imatinib‐treated *N* = 163; matched nilotinib‐treated patients *N* = 163; imatinib unmatched *N* = 658 and all imatinib‐treated patients (*N* = 821).

The probability of 5‐year OS was 90.5% (95% CI: 85.2–95.7) in imatinib‐matched patients, 94.4% (95% CI: 90.5–98.2) in nilotinib patients, and 86.3% (95% CI: 83.1–89.5) in imatinib‐unmatched patients. There was no statistically significant difference in survival between matched patients (*p* = 0.602), but a significant difference was observed among all groups (*p* = 0.021) (Figure 2A). When evaluating CML‐related death and its treatment (disease‐specific survival; DSS), there was no difference in survival between the imatinib‐matched and the nilotinib cohorts, nor was there a difference between any of the groups evaluated (*p* = 0.745; *p* = 0.809, respectively) (Figure S1A).

The probability of PFS at 5 years is illustrated in Figure 2B. In the imatinib‐matched group, it was 88.0% (95% CI: 82.4–93.7), while in the nilotinib group, it reached 92.9% (95% CI: 88.8–97.0). The imatinib‐unmatched group showed a PFS of 85.0% (95% CI: 81.7–88.3). There was no apparent treatment advantage for nilotinib among the matched cohorts, nor was there a significant difference between all the groups evaluated (*p* = 0.614; *p* = 0.053, respectively).

The estimated 5‐year event‐free survival (EFS) in each group was as follows: imatinib‐matched 53.5% (95% CI: 44.7–63.9) versus nilotinib 71.7% (95% CI: 63.8–79.7) versus imatinib‐unmatched 52.0% (95% CI: 49.5–59.1). A statistically significant difference was observed between the matched and all assessed groups (*p* = 0.025 and *p* = 0.029, respectively) (Figure S1B). Very similar results were demonstrated in the 5‐year failure‐free survival (FFS) analysis (*p* = 0.040 and *p* = 0.006, respectively) (Figure S1C).

No statistically significant differences were observed in the assessment of alternative treatment‐free survival (ATFS) between the matched and all groups (*p* = 0.487; *p* = 0.160, respectively) (Figure S1D). Similarly, the current leukemia‐free survival (CLFS) analysis showed no statistical significance in frontline therapy between the cohorts (*p* > 0.999; *p* = 0.103, respectively).

### 3.4 Safety of treatment in the matched cohorts

The overall incidence of hematological toxicity of all grades was comparable between the imatinib‐matched group (*n* = 163) and the nilotinib group (*n* = 163). The total number of cases with hematological toxicities of all grades was 79 of 163 (48.5%) in the imatinib cohort and 71 of 163 (43.6%) in the nilotinib group. The incidence of anemia and neutropenia was slightly higher in patients treated with imatinib compared to those treated with nilotinib (anemia 29.4% vs. 23.3%; neutropenia 27.6% vs. 12.3%, respectively). In contrast, the development of thrombocytopenia was more common during nilotinib treatment (27.0% vs. 35.0%). The development of grade 3/4 hematological toxicity was very low in both cohorts (Table S1).

The most common manifestations of non‐hematological toxicity of all grades in imatinib‐treated patients included muscle cramps (14.1%), peripheral edema (14.1%), musculoskeletal pain (11.7%), nausea and vomiting (10.4%), fatigue (10.4%), and skin rash (9.2%). In the nilotinib group, the most frequent symptoms included skin rash (all grades, 22.1%), fatigue (15.3%), muscle cramps (9.8%), muscle pain (9.8%), infections (8.0%), headache (6.7%), and nausea and vomiting (6.1%) (Table S1).

Cardiovascular toxicity was noted in six patients treated with nilotinib (3.7%) and in one patient in the imatinib‐matched group (0.6%). Only one patient had diabetes mellitus at the start of nilotinib, and no other risk factors were present. The median age of patients with a cardiovascular event was 55 years (52–80 years), and the median time to the development of these events was 15 months (11–75 months). During nilotinib treatment, hypercholesterolemia occurred in 3/6 patients, and hyperglycemia in 5/6 patients. Cerebrovascular toxicity was present in five patients (3.1%) in the nilotinib group and did not occur in any imatinib‐matched patient. This cardiovascular and cerebrovascular toxicity was not fatal in either case.

The INFINITY registry also monitors laboratory adverse drug reactions, which are more common in patients treated with nilotinib. Patients were most likely to develop hyperglycemia (22.1% for imatinib‐matched and 37.4% for nilotinib). However, this incidence is inflated by the fact that some patients were not fasting. Other common laboratory abnormalities included elevation of liver function tests, which was more common with nilotinib treatment compared to the incidence with imatinib (alanine aminotransferase elevation, 23.9% vs. 6.7%; gamma‐glutamyl transferase elevation, 23.3% vs. 2.5%; bilirubin elevation, 15.3% vs. 10.4%, respectively). As expected, elevation of cholesterol and triglyceride levels was also observed more frequently in the nilotinib group compared with the imatinib‐matched group (18.4% vs. 6.1% and 9.2% vs. 2.5%, respectively) (Table S1).

## 4 DISCUSSION

However, many questions related to the optimal management of newly diagnosed CML patients remain unanswered, and it is important to obtain additional results from routine clinical practice. Our analysis represents an evaluation of a large cohort of patients treated with first‐line nilotinib in real‐world practice. We found that nilotinib treatment was preferred by physicians, particularly in patients with high‐risk disease, without comorbidities, and who tended to be younger in age. This is consistent with recommendations for CML treatment.^15^, ^16^, ^17^, ^18^

Thus, the proportion of patients with CML‐CP treated with first‐line nilotinib in the INFINITY registry was only 16.6% (163 patients). The median age of patients treated with first‐line nilotinib was 46 years (range, 18–80 years), which aligns with the median age of 47 years in the ENESTnd study.^10^, ^11^ This is also in agreement with other published papers by Gugliotta and Milojkovic (46 and 51 years).^12^, ^41^ The representation of patients with high‐risk ELTS scores was slightly more frequent in the nilotinib‐treated group (19.0% nilotinib vs. 15.2% of all imatinib patients). The proportion of high‐risk Sokal scores was more dominant in the nilotinib group (27.0% nilotinib vs. 19.9% of all imatinib patients). This occurrence is identical to data from the ENESTnd study (28%).^10^, ^11^

In the imatinib‐matched group, 79 of 163 patients (48.5%) required a change of TKIs. The proportion of patients who switched to nilotinib was similar (75 of 163 patients; 46.0%). This observation agrees with data from the 2020 Milojkovic paper (45% changes in imatinib, 41% in 2GTKIs) or the 2021 Canet paper (41% imatinib, 36% in 2GTKIs).^41^, ^42^ However, major differences were found in the cause of the TKIs change in the evaluated groups. Patients on imatinib therapy were more likely to require a treatment change due to failure compared to the nilotinib group (39 of 163; 23.9% vs. 23 of 163; 14.1%, respectively). In contrast, intolerance or toxicity was the main cause of the TKIs change in the nilotinib group (imatinib 17 of 163; 10.5% vs. nilotinib 23 of 163; 19.6%, respectively).^11^, ^41^, ^42^

Adverse effects of TKIs are an important factor in the success of patient treatment, as their occurrence and severity can significantly affect patient compliance, which can have a subsequent impact on achieving a treatment response. The incidence of hematological toxicity was similar in both groups evaluated, and the incidence of grade 3/4 toxicity was less frequent in our patients than in the ENESTnd study.^10^, ^11^ The spectrum of non‐hematological adverse events was different in the two groups, as expected given the different drug profiles.

Despite the selection of patients for nilotinib treatment, cardiovascular events occurred in six patients (3.7%) and were suspected to be related to nilotinib. An increased number of cardiovascular events in CML patients treated with nilotinib as the first‐line was demonstrated in the 5‐year follow‐up of the ENESTnd clinical trial (arm with nilotinib 400 mg twice per day 15.9%; 300 mg twice per day 2.5%). It is essential to screen patients for vascular risk factors, such as hypertension, hypercholesterolemia, diabetes mellitus, or dyslipidemia, prior to starting nilotinib and to maintain follow‐up during treatment.

Treatment responses confirmed that patients treated with nilotinib achieved CCyR and MMR faster than those treated with first‐line imatinib. In patients on nilotinib treatment, MMR, MR4.0, MR4.5, and deeper responses were achieved at 5 years in 84.2%, 43.9%, and 22.8%, respectively. The number of patients achieving MMR was even slightly higher in our cohort than in the ENESTnd study in the NILO 2 × 300 mg daily arm (84.2% vs. 77.7%). However, the proportion of MR4.0 and MR4.5 responses was slightly lower in our cohort compared to the ENESTnd study results (43.9% vs. 65.6%; 22.8% vs. 53.5%).^10^, ^11^ This finding confirms the suitability of 2GTKI treatments for patients where we want to achieve treatment‐free remission.

In our propensity score analysis, we found that first‐line nilotinib treatment did not confer a benefit on 5‐year OS between the matched imatinib and nilotinib groups (90.5% vs. 94.4%). On the contrary, we would like to point out, that patients in the imatinib‐unmatched group had the worst survival (86.3%). This was likely influenced by the older age, comorbidities, and worse general condition of the patients in this group. Furthermore, considering the CML‐related deaths separately from those due to other causes, no difference was detected between all cohorts. It has been previously confirmed that patients with comorbidities benefit from imatinib treatment, and comorbidities do not affect the achievement of treatment responses, although they do have a negative impact on OS.^43^, ^44^, ^45^, ^46^ Nowadays, older age should not be a limiting factor for starting treatment with TKIs.^47^, ^48^, ^49^

Interestingly, in the nilotinib cohort, death from disease progression to advanced stages of CML occurred in 7/12 patients (58.3%), whereas in the imatinib‐matched group, disease progression was observed in only 3/16 patients (18.8%). Consistent with the results of the Italian Campus study, our findings suggest that first‐line nilotinib treatment does not fully eliminate the risk of CML progression, including deaths from disease progression.^50^ This is further supported by our PFS analysis, which shows no significant benefit of first‐line nilotinib, although there is a slight trend towards an advantage of initial nilotinib treatment (imatinib‐matched 88.0% vs. nilotinib 92.9%, respectively). These results are comparable to the published results of the ENESTnd trial, which also failed to confirm an advantage of first‐line nilotinib treatment on OS and PFS at 5 or 10 years (imatinib 91.8% and 91.2% vs. nilotinib 300 mg arm 93.7% and 92.3% at 5 years, respectively).^10^, ^11^ However, we did demonstrate a statistically significant difference in the impact of the chosen initial treatment when assessing EFS and FFS at 5 years. In the imatinib‐matched group, these rates were 54.3% and 53.5%, respectively, versus 71.7% and 71.7% in the nilotinib cohort. It is possible that the faster CCyR and MMR achieved with nilotinib treatment contribute to this difference.

In conclusion, the results of our retrospective analysis confirmed that imatinib is still the preferred choice of TKIs in the Czech Republic for the first‐line treatment of CML‐CP, especially for older patients with comorbidities, and, at the same time, an early change of imatinib in case of failure is preferred. Nilotinib treatment was started by physicians in younger patients with high‐risk disease without severe comorbidities. Our retrospective analysis from real‐world practice demonstrated a faster and deeper achievement of molecular response with nilotinib in frontline therapy. However, we did not confirm the superiority of nilotinib treatment on OS and PFS of patients.

## AUTHOR CONTRIBUTIONS

**Petra Belohlavkova:** Conceptualization (lead); data curation (lead); investigation (lead); methodology (lead); resources (equal); writing – original draft (lead). **Daniela Zackova:** Investigation (supporting); resources (equal). **Hana Klamova:** Investigation (supporting); resources (equal). **Edgar Faber:** Investigation (supporting); resources (equal). **Michal Karas:** Investigation (supporting); resources (equal). **Lukas Stejskal:** Investigation (supporting); resources (equal). **Eduard Cmunt:** Investigation (supporting); resources (equal). **Olga Cerna:** Investigation (supporting); resources (equal). **Ivana Jeziskova:** Investigation (supporting); resources (supporting). **Katerina Machova Polakova:** Investigation (supporting); resources (supporting). **Pavel Zak:** Investigation (supporting). **Tereza Jurkova:** Data curation (lead); formal analysis (lead); methodology (equal); software (lead); validation (lead); writing – original draft (supporting). **Marika Chrapava:** Formal analysis (supporting); software (supporting); validation (supporting). **Jiri Mayer:** Conceptualization (lead); data curation (lead); investigation (lead); methodology (lead); resources (equal); supervision (lead); writing – original draft (equal).

## FUNDING INFORMATION

This work was supported by DRO (UHHK, 00179906) and the Cooperatio Program. The project National Institute for Cancer Research (Programme EXCELES, ID Project No. LX22NPO5102) is funded by the European Union—Next Generation EU.

## CONFLICT OF INTEREST STATEMENT

The authors declare no conflict of interest.

## ETHICS STATEMENT

The clinical study was approved by the Health Research Ethics Board of Masaryk University Brno (01‐090224/EK).

## Supporting information


Figure S1A.

Figure S1B.

Figure S1C.

Figure S1D.

Table S1.

## Data Availability

Data are available at investigator upon request.
