# Supplementary figures and images for: Clinical efficacy and safety of first‐line nilotinib or imatinib therapy in patients with chronic myeloid leukemia—Nationwide real life data
Source: Cancer Med. 2024 Sep 13;13(17):e70158. doi: 10.1002/cam4.70158 (PMC11393449; doi:10.1002/cam4.70158)

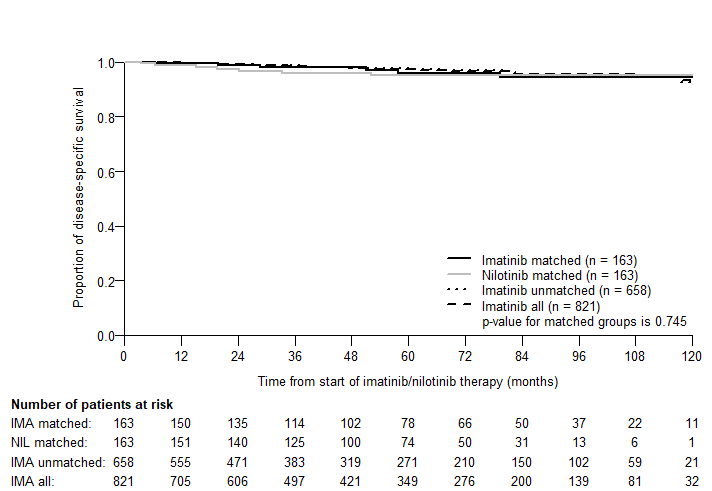

Supplement: Supplementary file 1 — Figure S1A. [file CAM4-13-e70158-s004.tiff]

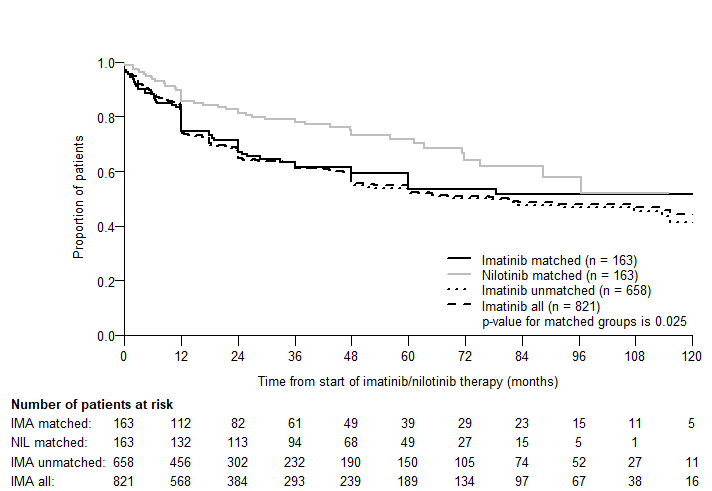

Supplement: Supplementary file 2 — Figure S1B. [file CAM4-13-e70158-s001.tiff]

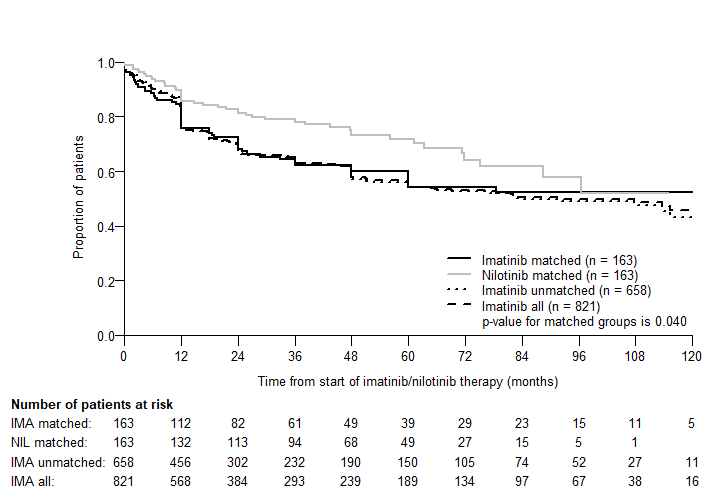

Supplement: Supplementary file 3 — Figure S1C. [file CAM4-13-e70158-s005.tiff]

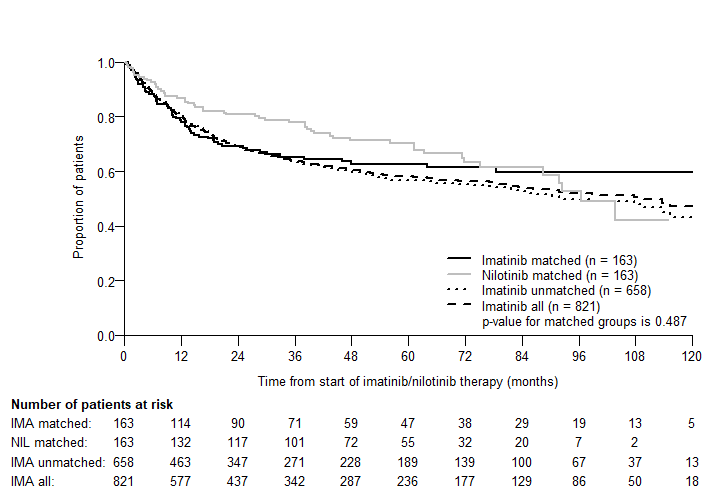

Supplement: Supplementary file 4 — Figure S1D. [file CAM4-13-e70158-s003.tiff]
