# Supplementary material for: Clinical efficacy and safety of first‐line nilotinib or imatinib therapy in patients with chronic myeloid leukemia—Nationwide real life data
Source: Cancer Med. 2024 Sep 13;13(17):e70158. doi: 10.1002/cam4.70158 (PMC11393449; doi:10.1002/cam4.70158)
Supplement: Supplementary file 5 — Table S1. [file CAM4-13-e70158-s002.docx]

Supplementary Table 1: Adverse events profiles in matched cohorts

| Adverse events | Imatinib  All grades (n, %) | Imatinib  Grade 3,4 (n, %) | Nilotinib  All grades (n, %) | Nilotinib  Grade 3,4 (n, %) |
| --- | --- | --- | --- | --- |
| Hematological toxicities  Anemia  Thrombocytopenia  Neutropenia | 79 (48.5%)  48 (29.4%)  44 (27.0%)  45 (27.6%) | 12 (7.4%)  0 (0.0%)  5 (3.1%)  8 (4.9%) | 71 (43.6%)  38 (23.3%)  57 (35.0%)  20 (12.3%) | 12 (7.4%)  1 (0.6%)  10 (6.1%)  4 (2.5%) |
| Non-hematological toxicities (occurring in ≥ 2% of patients)  Skin rash  Fatigue  Muscle cramps  Musculoskeletal pain  Peripheral oedemas  Nausea and vomiting  Infection  Diarrhoea  Headache  Hair loss  Conjunctivitis  Arthralgia  Dyspepsia  Cardiovascular event  Cerebrovascular event  Itching of the skin / pruritus  Fevers  Stomachache  Weight gain | 15 (9.2%)  17 (10.4%)  23 (14.1%)  19 (11.7%)  23 (14.1%)  17 (10.4%)  12 (7.4%)  13 (8.0%)  2 (1.2%)  2 (1.2%)  9 (5.5%)  3 (1.8%)  7 (4.3%)  1 (0.6%)  0 (0.0%)  2 (1.2%)  3 (1.8%)  2 (1.2%)  3 (1.8%) | 0 (0.0%)  0 (0.0%)  2 (1.2%)  2 (1.2%)  1 (0.6%)  2 (1.2%)  0 (0.0%)  0 (0.0%)  0 (0.0%)  0 (0.0%)  0 (0.0%)  1 (0.6%)  0 (0.0%)  0 (0.0%)  0 (0.0%)  0 (0.0%)  0 (0.0%)  0 (0.0%)  0 (0.0%) | 36 (22.1%)  25 (15.3%)  16 (9.8%)  16 (9.8%)  5 (3.1%)  10 (6.1%)  13 (8.0%)  1 (0.6%)  11 (6.7%)  10 (6.1%)  2 (1.2%)  7 (4.3%)  3 (1.8%)  6 (3.7%)  5 (3.1%)  3 (1.8%)  1 (0.6%)  2 (1.2%)  1 (0.6%) | 2 (1.2%)  1 (0.6%)  0 (0.0%)  1 (0.6%)  0 (0.0%)  1 (0.6%)  0 (0.0%)  0 (0.0%)  1 (0.6%)  0 (0.0%)  0 (0.0%)  2 (1.2%)  0 (0.0%)  3 (1.8%)  4 (2.5%)  0 (0.0%)  0 (0.0%)  0 (0.0%)  0 (0.0%) |
| Laboratory adverse events (occurring in ≥ 5% of patients)  Hyperglycemia*  ALT increase  GGT increase  Bilirubin increase  Hyperuricemia  Hypercholesterolemia  AST increase  Hypophosphatemia  Creatinin increase  ALP increased  Hypertriglyceridemia  Hypercalemia  Hypocalemia  Hyponatremia  Hypocalcemia  Lipase increase | 36 (22.1%)  9 (5.5%)  11 (6.7%)  4 (2.5%)  17 (10.4%)  5 (3.1%)  10 (6.1%)  15 (9.2%)  20 (12.3%)  14 (8.6%)  6 (3.7%)  4 (2.5%)  9 (5.5%)  3 (1.8%)  4 (2.5%)  1 (0.6%) | 0 (0.0%)  3 (1.8%)  1 (0.6%)  0 (0.0%)  2 (1.2%)  0 (0.0%)  2 (1.2%)  7 (4.3%)  0 (0.0%)  0 (0.0%)  0 (0.0%)  0 (0.0%)  1 (0.6%)  0 (0.0%)  1 (0.6%)  0 (0.0%) | 61 (37.4%)  44 (27.0%)  39 (23.9%)  38 (23.3%)  25 (15.3%)  35 (21.5%)  30 (18.4%)  20 (12.3%)  15 (9.2%)  18 (11.0%)  16 (9.8%)  15 (9.2%)  8 (4.9%)  13 (8.0%)  8 (4.9%)  10 (6.1%) | 2 (1.2%)  2 (1.2%)  2 (1.2%)  3 (1.8%)  1 (0.6%)  0 (0.0%)  2 (1.2%)  7 (4.3%)  0 (0.0%)  0 (0.0%)  3 (1.8%)  3 (1.8%)  1 (0.6%)  1 (0.6%)  0 (0.0%)  2 (1.2%) |

* majority of patients were not fasting

Abbreviations: ALT, alanine aminotrasferase; GGT, gamma-glutamyl transferase; AST, aspartate aminotrasferase; ALP, alkaline phosphatase
